# Significant improvement in nerve conduction, arm length, and upper extremity function after intraoperative electrical stimulation, neurolysis, and biceps tendon lengthening in obstetric brachial plexus patients

**DOI:** 10.1186/s13018-015-0191-y

**Published:** 2015-04-19

**Authors:** Rahul K Nath, Chandra Somasundaram

**Affiliations:** Texas Nerve and Paralysis Institute, 6400, Fannin Street, Houston, TX 77030 USA

**Keywords:** Elbow flexion contracture, Limb length discrepancy, Biceps tendon lengthening, Intraoperative electrical stimulation, Neurolysis, Obstetric brachial plexus injury

## Abstract

**Background:**

Progressive loss of extension and concomitant bony deformity of the elbow are results of persistent biceps contracture in obstetric brachial plexus injury (OBPI) patients, if they do not fully recover. This adversely affects the growth and development and functions of the upper extremity.

**Patients and method:**

We have performed biceps tendon lengthening (BTL) using a Z-plasty technique on OBPI patients aged 4 years to adulthood, who had been diagnosed with biceps tendon fixed flexion contractures. Ulnar, radial, and median nerve decompression was also performed at the same sitting. Somatosensory evoked potential (SSEP) monitoring was performed by stimulating the median and ulnar nerves at the wrist and the radial nerve over the dorsum of the hand and recording the peripheral, cervical, and cortical responses.

Seven children with obstetric brachial plexus palsy with an average age of 11 years (8.7–14.2 years) were included in this report. Mean follow-up time was 7.4 months (4–11 months). All the patients in this report had the elbow flexion contractures greater than 30°.

**Results:**

Mean flexion contracture was 35° (30°–45°) preoperatively, which was improved to 0°–10° postoperatively with an average follow-up of 7 (4–11) months. This surgical procedure corrected the elbow flexion contractures, about an average of 25° and an improved length almost to normal, and improved the upper extremity functions. Neurophysiological data showed significant improvement in conduction of all three nerves tested after neurolysis. Further, median and radial nerve amplitude increase was statistically significant.

**Conclusion:**

Statistically significant improvement in biceps length as well as nerve conduction was observed after the surgery. None of the children in our study lost biceps function, although weakness of the biceps is both a short- and long-term risk associated with biceps lengthening.

## Background

Limb length discrepancy and flexion contractures of the elbow are common occurrence, limiting functions of the forearm and hand in permanent obstetric brachial plexus injury (OBPI) patients [1-3]. The flexed elbow posture not only limits upper extremity functions but also may cause pain. The reported prevalence of elbow flexion contracture (EFC) in OBPI patients is ranging widely between 4.6% and 89.5% [1,4-6]. EFCs are reported also in children with cerebral palsy involving the upper extremity [7]. Several authors have proposed different etiologies for the occurrence EFC in OBPI.

Hoffer and Phipps (2010) [8] suggested that EFC may be caused by deformation of the elbow joint or imbalance of the muscles or a combination of both [8]. Insufficient power (<M4) of the triceps was reported to cause flexion contracture of the elbow [9]. EFC seems to be caused by brachialis muscle pathology [10], and this is not an osseous abnormality. Sheffler et al. [6] also found that majority of the OBPI patients with EFC have normal-appearing elbow radiograph and no radial head dislocation.

Further, they [6] reported the prevalence of contracture increased with increasing age and was found to increase by 4.4% per year before treatment. OBPI with EFC limited to 15° to 20° are usually managed by occupational therapy/long-term night splints. In some patients, the deficit can be severe (30°–80°), and this is treated surgically [1,10-14]. Standard surgical treatments reported are soft tissue release at the elbow joint in conjunction with lengthening of the biceps, brachialis, and flexor-pronator mass [12,15-19].

We have demonstrated previously that median nerve conduction and shoulder abduction were significantly improved after modified Quad surgery with neuroplasty, internal microneurolysis, and tetanic stimulation of the median nerve in OBPI patients [20]. Further, surgical decompression and neurolysis and tetanic electrical stimulation of the long thoracic nerve significantly improve scapular winging and the associated limitations on shoulder movement [21,22].

Here, we were able to achieve significant improvements in nerve conduction after neurolysis with tetanic stimulation of median, radial, and ulnar nerves in seven OBPI patients.

In addition, we report significant improvement in arm length after BTL surgery and contracture release. In this series of patients, who had >30° elbow flexion contracture and significantly shorter arm when compared to the unaffected arm.

## Patients and methods

Seven children with obstetric brachial plexus palsy with an average age of 11 years (8.7–14.2 years) were operated. Average follow-up time was 7.4 months (4–11 months). All patients had the elbow flexion contractures greater than 30°.

The patients underwent general anesthesia. The arm and hand were prepped and draped in the usual sterile fashion. An incision was created over the volar elbow crease to expose the underlying biceps tendon, the brachialis muscle fascia, and the median and radial nerves. The lacertus fibrosus was identified and released sharply. The biceps tendon was meticulously dissected proximally and distally then sharply released in a Z fashion, allowing improved passive extension of the elbow joint.

The radial and median nerves were found to be tethered and constricted within the connective tissue of the elbow, probably due to the lack of excursion related to the tendon contractures. Each nerve was externally neurolysed. Internal neurolysis was then performed on each nerve to improve finger and wrist flexion and extension. Similarly, the ulnar nerve was exposed, externally and then internally neurolysed.

The brachialis fascia was exposed, and a series of step-lengthening fascial releases were performed to lengthen the brachialis muscle (included in Z-plasty). Partial release of the collateral ligaments was also performed at this point.

### Intraoperative tetanic stimulation and neurophysiological evaluation

Somatosensory evoked potential (SSEP) monitoring was performed by stimulating the median and ulnar nerves at the wrist and the radial nerve over the dorsum of the hand and recording the peripheral, cervical, and cortical responses. Surgical decompression of all three nerves was performed. We evaluated the efficacy of intraoperative electrical stimulation-induced SSEPs in pre- and post-decompression during BTL surgery.

This was a retrospective study of patient charts, which exempted it from the need for IRB approval in the United States. Patients were treated ethically in compliance with the Helsinki declaration. Documented informed consent was obtained for all patients.

### Statistical analysis

Paired Student’s t-tests were conducted using Microsoft Excel 2003 with the Analyze-It plug-in (Redmond, WA; and Leeds, UK) to determine if differences between preoperative and postoperative SSEPS was statistically significant. The p values were two tailed and considered significant if less than or equal to 0.05.

## Results

**Figure 1:**
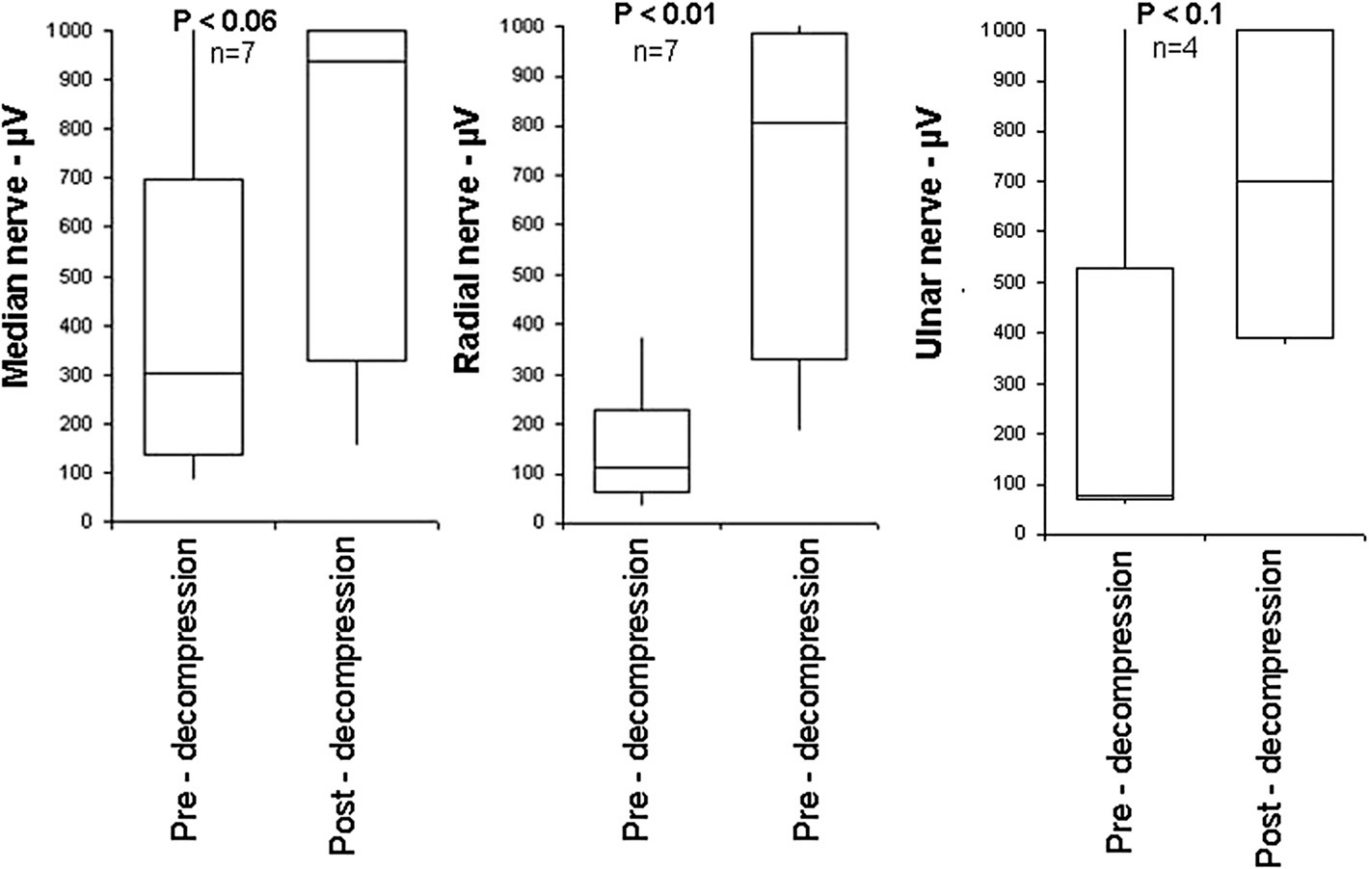
Nerve conduction (μV) after intraoperative electrical stimulation and monitoring before and after nerve (median, radial, and ulnar) decompression in OBPI patients. Nerve conduction was improved statistically significantly for both median and radial nerve.

Pre- and post-decompression revealed a reduction in stimulus threshold and an increase in amplitude for all three nerves (median, ulnar, and radial) tested after the procedure (Figure 1). Amplitude is the response of nerve stimulation that is usually measured in microvolts (μV). The stimulus threshold is the minimal stimulation intensity that elicits SSEP. Nerve conduction was improved statistically significantly for both the median and radial nerve. Of the seven patients, ulnar nerve conduction data was available for only four patients, and that might be the reason that ulnar nerve conduction was not statistically significant (Figure 1).

**Figure 2:**
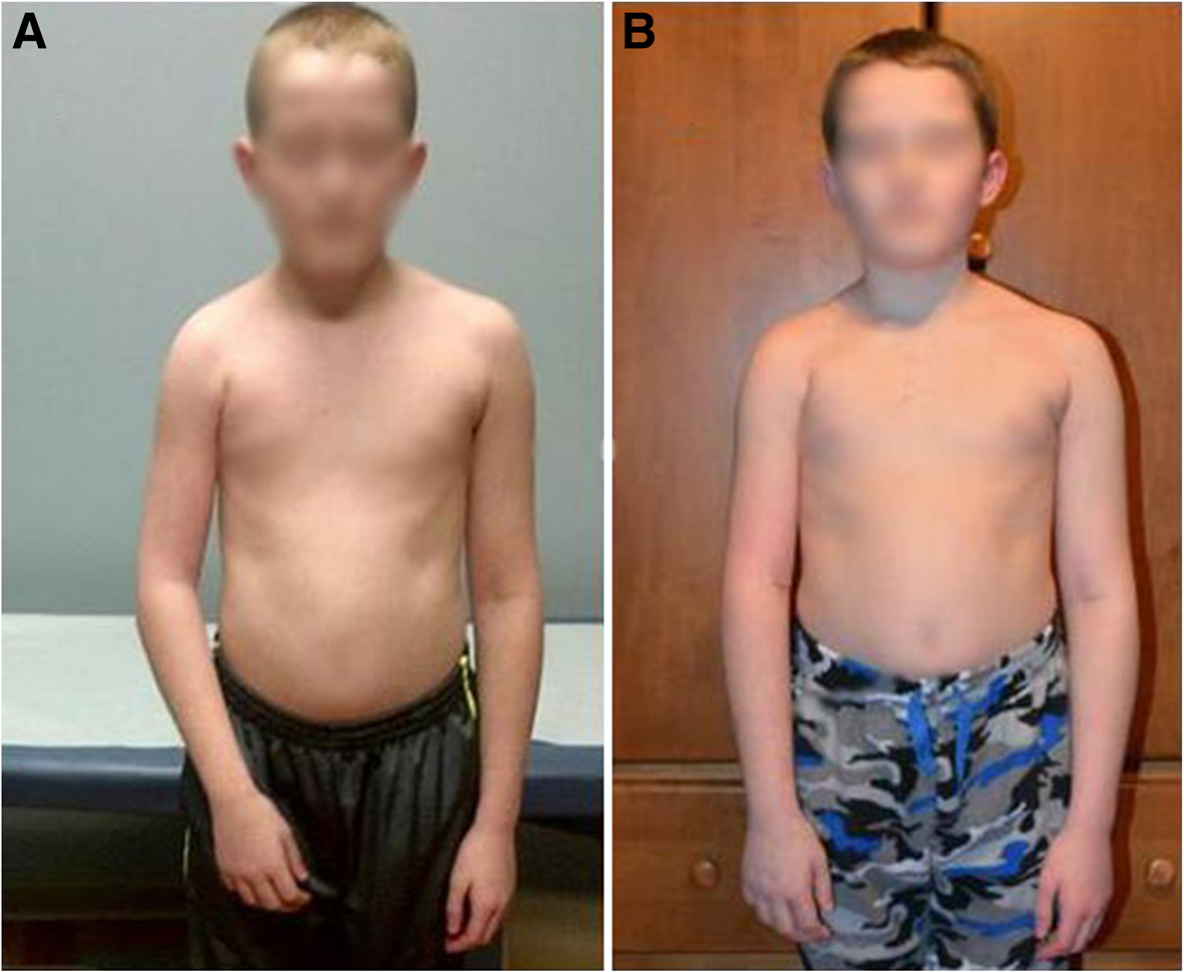
An OBPI male patient with elbow flexion contracture about 40° and shortening of the right arm before **(A)** and almost normal after **(B)** biceps tendon lengthening.

**Figure 3:**
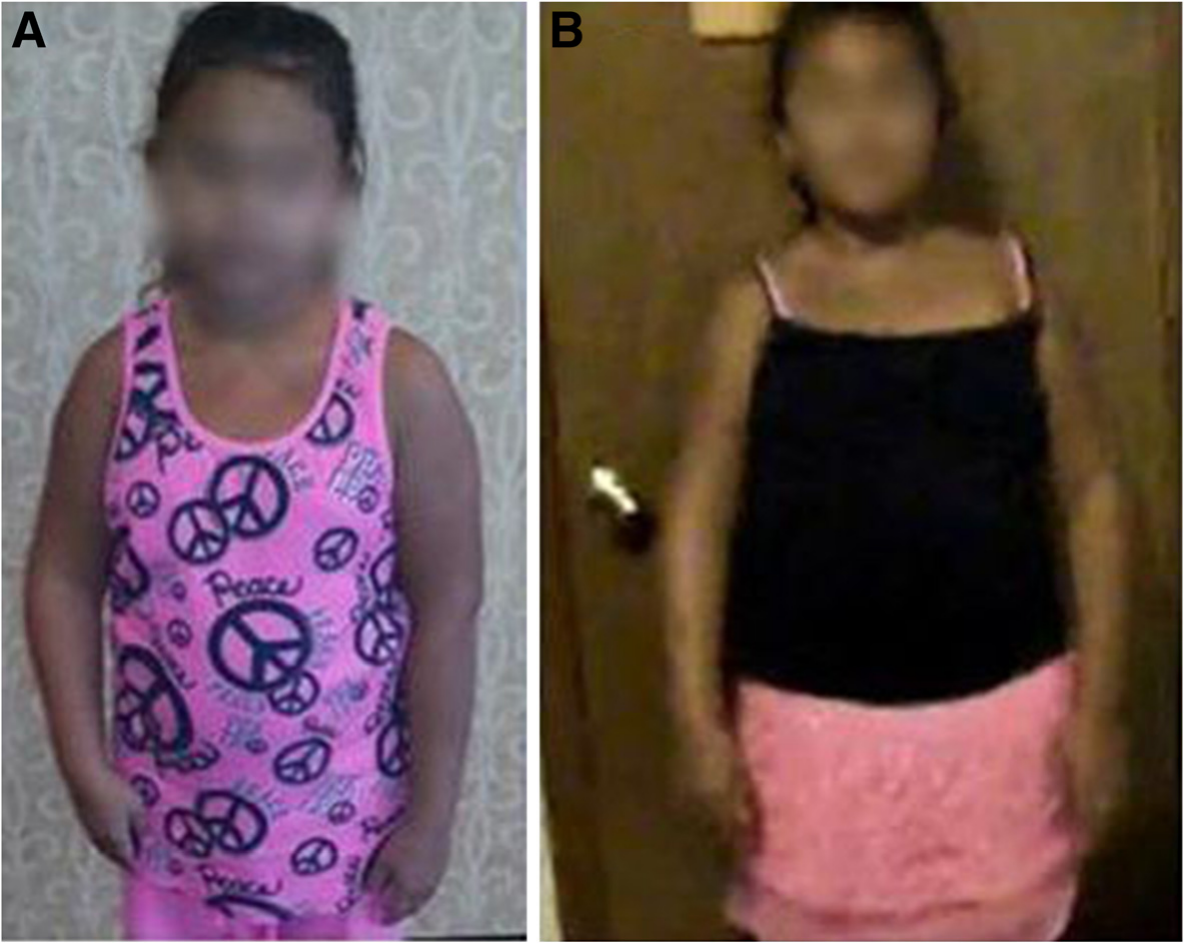
An OBPI female patient with elbow flexion contracture and shortening of the right arm before **(A)** and almost normal after **(B)** biceps tendon lengthening.

Mean flexion contracture was 35° (30°–45°) preoperatively, which was improved to 0°–10° postoperatively with an average follow-up of 7 (4–11) months (Table 1). We demonstrate here that the BTL surgery corrected the elbow flexion contracture, by an average of 25° and improved the arm length, and upper extremity functions (Figures 2 and 3). Further, there was no change in muscle strength in five patients and biceps strength improved in two patients after BTL surgery (Table 1).

**Table 1:** **Outcome of biceps tendon lengthening in OBPI patients**

**Nerve injury**	**Age at surgery**	**Strength preop**	**Strength postop**	**Elbow flexion contracture**		**Follow-up months**
				**Preop**	**Postop**	
Total	12.9	4+	4+	30°	10°	11
C5-7	12.2	3.5	4+	45°	20°	4
C5-7	9.5	4+	4+	-	-	6
C5-7	10.7	4+	4+	40°	0°	7
Total	9.6	3.0	3.0	30°	10°	7
C5-7	8.7	4.0	4.0	30°	10°	11
C5-6	14.2	3.5	4.0	45°	10°	6
*Mean*	*11.0*			*35°*	*10°*	*7*

The mean reduction of the elbow flexion contractures was 25° in patients with C5-C7, and 30° in total plexus injury after BTL surgery (Table 1). Our results show that the outcome of this surgery was not significantly different in terms of the severity of the injury in our study group.

## Discussion

We have previously demonstrated that neurolysis with intraoperative tetanic stimulation significantly improved median nerve conduction during modified Quad surgery [20] and an immediate effect on muscle recovery [21,22] in OBPI patients. Modified Quad [23] is a modification of the combination of muscles released and their insert positions to improve upon a previously described operation [24]. In modified Quad procedure, the latissimus dorsi, teres major, subscapularis, and pectoralis muscle contractures are released [23]. BTL surgery was performed for severe flexion contracture of the elbow. Neurolysis with tetanic stimulation of median, radial, and ulnar nerves was also done to improve this nerve conduction. In this report, intraoperative electrical monitoring confirmed radial, ulnar, and median nerve conduction deficits in these patients prior to decompression and BTL surgery.

Neurolysis with tetanic stimulation of median, radial, and ulnar nerves can have a significant impact on the elbow joint contractures caused by OBPI. Combined with BTL surgery and contracture release, we were able to achieve significant improvements in arm length and function in this series of patients.

All patients in our present study documented to have shoulder dystocia. None of them had Horner’s syndrome. Five of seven patients were delivered (documented) using either by vacuum or forceps or both. Of all seven patients, one was documented to have finger movement at birth, and no movement for other six patients at birth. All seven patients had the secondary muscle surgery (modified Quad). Six of seven patients also had the bony surgery, triangle tilt in addition to BTL surgery with us. One patient (seventh patient in Table 2) had primary nerve graft at another clinic before presenting to our institution. In addition, two patients had tendon transfer before having treatment with us, and one had forearm osteotomy at another clinic.

**Table 2:** **Patients demographics**

**Patient**	**Nerve injury**	**Birth history**	**Previous surgeries**	**Age at BTL surgery**
1	total	SD, no Horner’s syndrome, instruments used, nothing moved	TT, MQ	12.9
2	C5-7	SD, no Horner’s syndrome, instruments used, nothing moved	TT, MQ	12.2
3	C5-7	SD, no Horner’s syndrome, nothing moved	TT, tendon transfer Neurolysis	9.5
4	C5-7	SD, no Horner’s syndrome, fingers moved	TT, MQ, FO	10.7
5	Total	SD, no Horner’s syndrome, instruments used, nothing moved	TT, MQ, tendon transfer	9.6
6	C5-7	SD, no Horner’s syndrome, nothing moved	TT, MQ	8.7
7	C5-7	SD, no Horner’s syndrome, instruments used, nothing moved	MQ, nerve graft neurolysis	14.2

SD shoulder dystocia, TT triangle tilt, MQ modified Quad.

Of seven patients, five had C5-C7 upper plexus injury, and two had total brachial plexus injury (Table 2). The mean severity of the contractures in C5-C7 patients was 35° (30° to 45°) and 30° in total plexus injury patients (Table 1). This indicates that the extent of the contractures is not significantly associated with the severity of brachial plexus injury as reported in the literature [6].

The outcome of the surgery and the severity of the contractures in our patients with C5-C7 and total brachial plexus injury were also not significantly different (Table 1) as reported in the literature [6]. In addition, we found that the extent of the contracture was higher (40°–45°) in adolescent and teenage (10.7, 12.2, and 14.2 year old) patients, when compared to the younger children (30° in 8.7, 9.5, and 9.6 year old) except for one patient in our study group (Table 1). Further, elbow position sense is also found impaired in older children and adolescents with OBPI [25].

We demonstrated here that the biceps tendon lengthening corrected the elbow flexion contracture, at about an average of 25° and improved the arm length significantly, and therefore the upper extremity functions.

## Conclusion

Statistically significant improvement in biceps length as well as nerve conduction was observed after the surgery. None of the children in our report has lost biceps function, although weakness of the biceps is both a short- and long-term risk associated with biceps lengthening. We have not noticed recurrences of nerve compression or elbow flexion. We are currently studying the recovery of the three types of nerve compression but do not have data currently. The limitation of our manuscript is the lack of control group and the comparison with nonoperative treatment of serial casting and nighttime splinting.

### Ethical approval statement

Written informed consent was obtained from all patients for publication and accompanying images. A copy of the written consent is available for review on request.
